# Premature Death, Underlying Reasons, and Preventive Experiences in Iran: A Narrative Review

**DOI:** 10.34172/aim.2023.61

**Published:** 2023-07-01

**Authors:** Iman Razeghian-Jahromi, Yasin Ghasemi Mianrood, Mahintaj Dara, Pouria Azami

**Affiliations:** ^1^Cardiovascular Research Center, Shiraz University of Medical Sciences, Shiraz, Iran; ^2^Stem Cells Technology Research Center, Shiraz University of Medical Sciences, Shiraz, Iran

**Keywords:** Cardiovascular disease, Non-communicable disease, PolyIran, Polypill, Premature mortality

## Abstract

Premature mortality (PM) has emerged as a global health challenge. This is of eminent importance in low- and middle-income countries, where nearly three fourths of the deaths occur. The concerning issue is the early occurrence of fatal events in productive age. Fatal events before the age of 70 are called PM, which mainly result from cardiovascular diseases (CVDs). Iran as a middle– income country greatly suffers from the cardiovascular burden, which accounts for almost 50% of all PM. Despite substantial success in reducing mortality due to communicable diseases across different age ranges, urbanization and pervasiveness of cardiovascular risk factors have increased the death rate in adults in recent years. Undoubtedly, such lifestyles have imposed heavy costs on the healthcare system; it is possible that PM reduction, as one of the fundamental elements of sustainable development goals defined by the World Health Organization (WHO), would not be reached by the due date. Recently, researchers have introduced a cost-effective fixed-dose drug combination, the so-called polypill, in order to attenuate the detrimental effects of hypertension and hyperlipidemia, as two strong cardiovascular risk factors. PolyIran and PolyIran-Liver studies are two pivotal clinical trials that revealed the feasibility of primary and secondary prevention of premature cardiovascular mortality, both in an urban and a rural population. In the present narrative review, we tried to present a comprehensive appraisal on PM status, its underlying reasons, and the impact of polypill strategy on PM prevention in Iran.

## Introduction

 Among 17 universal goals, 169 targets, and 230 indicators defined by the World Health Organization (WHO) in the context of Sustainable development goals (SDGs) up to 2030, health is the core.^1^ Development of a healthcare system is determined by the rate of mortality.^2^ During the last decades, the consistent rise in global population has resulted in an increasing number of deaths irrespective of sex. Interestingly, the global life expectancy has also experienced an ascending trend simultaneously. In low-income countries, there were falls in the occurrence of certain diseases like diarrhea, lower respiratory infections, and neonatal fatality while in middle- and high-income countries, cardiovascular- and cancer-related mortality were decreased.^3^ In particular, life expectancy increased from 65.0 to 68.4 years during the period of 2000 to 2015 in the Eastern Mediterranean Region (EMR).^4^ Life expectancy shows mortality rate in a population in a given year, and demonstrates the trend of deaths over time.^5^

 Iran, as a middle-income country in the EMR region, has experienced remarkable transitions in the form of social, economic, and demographic changes over the last 40 years.^6^ Although its population increased from 58.5 million in 1990 to 84.3 million in 2019,^7^ the mortality rate was considerably reduced due to significant improvements in life sanitation and socioeconomic development. Thereby, life expectancy was elevated to 81.6 for men and 76.1 years for women in 2019, one of the highest in the region.^8^ Obviously, such an improvement is not shared equally between all individuals of the society. In other words, life expectancy shows the average mortality level, and this conceals variations in the length of life between individuals.^9^ Therefore, it is necessary to complement life expectancy with other indices in order to have a precise evaluation of the health condition of the society.

 In line with this notion, premature mortality (PM) emerged as an important health index, the reduction of which has been planned in the context of SDGs.^10^ According to GBD projection, premature cardiovascular disease (CVD) deaths will rise to 7.8 million by 2025.^11^ The WHO estimates that PM causes an economic loss of about US$ 7 trillion in low- and middle-income countries (LMICs) by 2030.^12^ PM is usually considered as the incidence of death under the age of 70,^13^ and can be a reflection of the underlying reasons of death at younger ages.^14^ Notably, the underlying reasons of PM vary widely across regions.^15^ Ischemic heart disease (IHD) and stroke are the major causes of PM throughout the world.^11^ These two in combination with cancer, respiratory disease, and diabetes constitute more than 80% of all PM.^16^

 The highest premature deaths related to CVD occur in the EMR followed by Southeast Asia.^17^ Currently, non-communicable diseases (NCDs) are the principle cause of PM, and about 86% of the global PM occurs in LMICs.^6^ Despite a dramatic decline in global CVD mortality rates, LMICs experience an increase in number of life years lost due to premature cardiovascular deaths. The higher incidence of PM in these countries in comparison to developed areas may be explained by dissimilar management and treatment of preclinical population and the diagnosed CVD patients.^17,18^ These facts simply demonstrate a remarkable and uniform shift in the nature of diseases from communicable to non-communicable in these countries.^19,20^ Unlike the inevitable nature of death itself, it is quite practical to avoid PM and extend life expectancy.^21^ Considering the importance of PM, its underlying causes, and efficient preventive strategies, there is paucity of data regarding these health concerns within the countries, especially in LMICs like Iran.^15^

## Premature Mortality in Iran

###  National Level

 In developed countries, about 80% of deaths occur during retirement. On the contrary, the majority of deaths in developing countries like Iran happen in active ages in terms of PM.^22^ PM inflicts enormous financial restrictions on the affected family, and imposes considerable economic burden on the society.^23^ Notably, the risk of mortality in the affected family is also increased upon premature death of a middle-aged member.^24,25^ In one study, it was reported that nearly 45% of deaths in Iran occurred in the ages of 30‒69 years though in a decreasing trend from 2006 to 2016. Within this age range, men had a higher probability of death due to all causes and NCDs.^6^ Although NCD-related deaths increased between 30‒69 years during 2006‒2016, the unconditional probability of dying due to NCDs experienced a decline (21.3% to 14.9%) in this age group. Except for diabetes, PM due to cancer, CVD, and chronic respiratory disease decreased. This was further confirmed by a WHO report and Global Burden of Disease study.^26,27^ It was projected that the decrease in PM will continue up to 2030, and Iran will reach the SDGs target in PM reduction.^6^ Consistent with this report, another study showed that the decreasing trend of all-cause mortality in all age groups and both sexes between 2006 and 2015 will continue during 2016‒2030, as well. Compared with 2010, the decline in mortality is more prominent in males in 2030 irrespective of age group. It means that the highest reduction would be in the male adults between 25‒29 years of age (52.4%) and females 60‒64 years of age will show the lowest reduction (2.2%).^2^

 However, other studies claim contradictory projections. One study in 2019 reported that there is a significant gap to reach the SDGs target of 11.6% as the share of PM in Iran by 2025.^28,29^ if the current trend maintains.^7^ Although the share of PM in 1990 (52.7% of all-age mortality) due to four principal NCDs (neoplasms, CVD, chronic respiratory diseases, and diabetes) decreased to 37.1% in 2019, Iranians died from NCDs 88.0% more in 2019 than in 1990. In 2019, in fact, about 54.6% of PM were due to the top-ranking NCDs.^7^

###  Subnational Level

 A study conducted in the capital of Iran, Tehran,^30^ showed the crude incidence rate of PM to be 3.15 per 1000 person-years while the age-standardized rate was estimated to be 1.90 and 2.35 per 1000 persons-year based on the Iranian and WHO standard population, respectively. Over a median follow-up of 13.84 years, 262 PM occurred (58.3% males). The incidence of CVD-related PM was reported to be 5.06 per 1000 person-years. CVD was diagnosed as the main cause of PM (48%) followed by cancer (19%).^30^ In another survey in one of the northern provinces of Iran, Golestan, 50,045 individuals aged ≥ 40 (95.0% were younger than 70 years) participated from 2004 to 2008.^31^ Of 6,347 deaths, 63% were premature, which were classified based on the causes: 50% for communicable, maternal, prenatal, and nutritional, 64% for NCDs, and 82% for injuries. IHD (33.9%) and stroke (14.0%) were identified as the leading causes of PM followed by cancers (9.2%) and road injuries (4.7%). In total, NCDs accounted for 88.8% of PM, and PM was known to be strongly associated with diabetes, opium consumption, and hypertension. To substantiate, diabetes increased the risk of death up to 139%. Interestingly, it was found that PM and socioeconomic factors were intimately intertwined. In the Golestan study, lack of wealth and education, which constitute the underlying causes of 37% of all PM, were stronger compared with any other individual risk factors. The authors interpreted that high PM in this population may be related to the fact that the participants were from villages and 70% of them were illiterate.^15^ Eight risk factors explain 76% and 69% of the population attributable factor (PAF) in males and females, respectively. Among PM, 7.0% were < 50 years, 30.5% were < 60 years, and 63.3% were < 70 years. It was estimated that people reach the age of 70 with a probability of 79.2%, if they survive up to the age of 40.^15^ The authors ultimately concluded that the rate of PM determined in this study is very similar to the average PM throughout Iran.^31^

 A counterfactual analysis of death registration data was carried out in the central part of Iran, Markazi province, during 2006‒2007.^32^ Among 7,176 total deaths, years of life lost (YLL) due to PM were 1.8 times greater in males. In the context of CVD, overweight for males besides obesity and overweight for females were found to be the most important reasons of YLL. Out of ten main causes, which composed 91% of the total burden, three including coronary heart disease, cancer, and accidents were responsible for the highest YLL due to PM. Indeed, it was revealed that the decrease in YLL due to CVD was faster in this province compared with the global pace.^32^ In another study in northwestern Iran, Zanjan province, the three major causes of PM were found to be CVD, unintentional injuries, and malignancies. YLL was 1.5 times higher in males in this study.^33^

 In another survey in the Hamadan province, western Iran, the first three leading causes of death were identified as myocardial infarction, cerebrovascular events, and transport accidents.^14^ With the highest mortality rate in 2006, Hamadan experienced a crude mortality rate of 5.60 per 1000.^34^ The first and second major causes of PM were diseases of the circulatory system and external causes of morbidity in both 2006 and 2010.^14^ The YPLL of the circulatory system disorder showed a decrease in 2010 compared to 2006. However, the external causes of morbidity increased during this period.^14^ These findings were repeated in another study conducted in six provinces throughout different geographical regions of Iran (Hormozgan, East Azerbaijan, Khorasan, Bushehr, Yazd, and Charmahal & Bakhtiary) during 2003. It was demonstrated that traffic injuries and diseases of the circulatory system were the first and second causes of PM.^35^ Another study showed that IHD and transport accidents were the two main causes of death in 2009.^34^ Eight NCDs, particularly IHD, cerebrovascular diseases, transport injuries, and intentional self-harm were introduced as the main culprits of PM in both 2006 and 2010 in another investigation.^14^

## Relationship Between PM and NCDs

 Economic stability of individuals, families, communities, and the whole world population has been undermined by NCDs.^36^ The aim of SDGs 3.4 is the reduction of premature deaths associated with NCDs throughout the globe including Iran.^37^ Nearly 80% of the world population live in developing countries, and the health of these huge habitants have been currently affected by NCDs.^14^ Even in some high-income countries, the decrease in CVD and the consequent increase in life expectancy have stopped or reversed in recent years.^38^

 While NCDs ranked 15^th^ in 1990,^39^ heart diseases alone will attain the first rank by 2030.^40^ This condition becomes a concerning matter when estimations revealed that 70% of deaths in developing countries were due to NCDs in 2020. Such an escalating growth may be related to epidemiological transition that inflicts a multitude of complications on the healthcare systems.^40,41^ The Institute for Health Metrics and Evaluation has issued a statement in 2019 in which IHD was known as the leading cause of total and premature death in Iran.^42^ NCDs will be the cause of about 80% of PM by 2030 in Iran.^37^

 Iran suffers from a high mortality rate due to CVD. Also, one of the highest age-standardized prevalence rates of CVD ( > 9000 cases per 100 000 person) belongs to Iran.^43,44^ About half of the 19 million CVD deaths in the world are premature, and half of the population with the life expectancy of 77 years in both sexes do not reach 70 years of age (unpublished data). A cohort study in the Isfahan province showed that the risk of CVD was higher in young individuals especially in women, which was contrary to the previous reports.^45^ Although the age-standardized death rates due to IHD and stroke declined dramatically (80%) at the global level,^46^ IHD and stroke maintained the first and second ranks of age-standardized death rate in both 1990 and 2019 in Iran.^7^ IHD solely accounts for approximately 26% of total deaths, which caused the highest disease burden in Iran.^47^ One study claimed that the rise in IHD incidence is greater among females.^45^ The disadvantageous sequels of IHD are more likely represented in reduction of life expectancy in Iranian females in comparison to their counterparts in neighboring countries.^48^ However, IHD mortality was found to be higher in males in another study.^4^ Also, the prevalence of stroke is higher in Iran than the Western countries.^49^ Indeed, morbidity and mortality of young adults are higher compared with their peers in Western countries.^50,51^ Due to the higher life expectancy in Iranian women, stroke was more prevalent in this gender.^52^

 In Iran, avoidable causes of death are significantly related to the increasing burden of inequality.^4^ The share of metabolic risk factors in all-cause health burden increased from 1990 to 2019.^7^ Compared with 43.0% in 1990, disability-adjusted life year (DALY) due to NCDs reached 78.1% in 2019. However, the age-standardized DALY rate due to NCDs greatly decreased by 25.9% from 1990 to 2019. The reduction in CVD burden had the highest influence (2.9 years) on the elevation of life expectancy.^7^ However, the increased total number of IHD deaths is the inevitable consequence of the rise in life expectancy and population growth.^53^ Even if the current levels of CVD risk factors remain constant, YLL due to CVD will increase drastically in the upcoming years.^54^

 Strong CVD risk factors such as hypertension, diabetes, high low-density lipoprotein cholesterol (LDL-C), low LDL-C, hypertriglyceridemia, hypercholesterolemia, obesity, and smoking are all modifiable and hence, up to 80% of CVD outcomes are preventable.^45^ Among risk factors, hypertension plays a more pronounced role than the other ones in raising the risk of CVD events in Iran.^55^ However, PM is generally associated with diabetes, smoking, family history of premature CVD, and prevalence of CVD, as well. More than 40% of PM result from diabetes, hypertension and current smoking, and the most pronounced PAF is attributed to diabetes.^30^ The risk of PM was higher in diabetic patients compared with non-diabetic individuals,^56^ and this risk becomes prominent in younger patients.^57^ The risk of PM is lower in females. Paradoxically, adults with overweight and obesity experienced lower risk.^30^ Even more, this so-called obesity paradox is maintained by considering different obesity indices, either general adiposity (body mass index) or central adiposity (waist circumference).^58^ The possible explanation for this observation is that Iranians who have a moderately increased body fat hold more metabolic reserves, and usually refer to medical care centers sooner. This may be associated with earlier receiving of preventive and therapeutic interventions.^59^

## Primary Prevention of PM: The Polypill Approach

 Policymakers and health authorities have encountered serious hurdles for responding to the contemporary threats in terms of prevention and control. In this regard, making appropriate decisions at the population level should be based on the burden of disease estimates and health concerns.^60^ Designing global-level and context-specific strategies for reduction of premature CVD mortality needs an updated information about this type of death. In order to achieve a one-third reduction in the rate of NCD-related deaths, the current annual decrease of 1.1% from 2010‒2015 should be doubled from 2015‒2030. Notably, this global target mainly depends on prevention and control of CVD.^61^

 In 2003, a modeling analysis proposed that a fixed-dose combination of medications decrease disease burden by 80% in adults ≥ 55 years of age with atherosclerotic CVD.^62^ Regarding primary prevention, trial-based evidence about the effects of this regimen on clinical outcomes should be explored in order to resolve some debates around this so-called polypill.^63^ Primary prevention is defined by those interventions that postpone the onset of a disease.^64^ As the majority of CVD clinical outcomes happen in individuals without a history of vascular disease,^65^ primary prevention is vital for attenuating the risk of life-threatening events at the population level. It was shown that lowering LDL cholesterol and blood pressure via pharmacotherapy is an efficient strategy for reducing cardiovascular events both in individuals with and without vascular disease.^66-68^ However, prescription of such medications is not usual for primary prevention.^65^

 A fixed-dose combination of aspirin, statins, and antihypertensive drugs was nominated as one of the optimal choices for this aim.^69^ The efficacy of this approach has been examined in a meta-analysis including three large randomized controlled trials up to date (TIPS-3, HOPE-3, and PolyIran).^65^ It was shown that this intervention reduced the risk of CVD by 38%, and was mostly useful in lowering the incidence of myocardial infarction, revascularization, and stroke. The promising point of polypill is rapid preventive decline in fatal and non-fatal cardiovascular events. The benefit of this treatment became apparent within one year after use and improved in the following years. Individuals receive the beneficial effects of polypill irrespective of presence of comorbidities like hypertension, diabetes, or increased LDL cholesterol, and this simply shows the comprehensive applicability of polypill administration across a wide range of people with different profiles of risk factors. Moreover, the advantageous consequences of polypill are mainly delivered to older adults.^65^

 The PolyIran study, as the first large-scale, long-term, and pragmatic randomized trial in Iran, investigated the effect of once-daily four-component polypill (aspirin, enalapril/valsartan, atorvastatin, and hydrochlorothiazide) on primary and secondary prevention of major cardiovascular events in individuals from rural regions.^70^ Polypill reduced the risk of fatal and non-fatal IHD and stroke by 40% during a follow-up period of five years with no additional adverse events. Indeed, high adherence or longer use of the polypill resulted in greater risk reduction. Interestingly, risk reduction was consistent irrespective of age, gender, smoking, and preexisting hypertension/high blood cholesterol/CVD. Like the HOPE-3 study,^71^ PolyIran demonstrated that a fixed-dose combination can be used as a low-cost primary prevention approach, especially in LMICs. The polypill-based intervention helps to achieve the SDG target in lowering CVD-related PM by at least a third before 2030.^70^

 The PolyIran-Liver study, an extension of the PolyIran study, is the first trial that investigated the effects of polypill on an urban population, who suffer from fatty liver or non-alcoholic steohepatitis.^72^ The results showed that polypill is safe, well-tolerated, and might be effective in reducing the risk of major adverse cardiovascular events (MCVE) in people > 50 years of age. The findings of the PolyIran-Liver study were similar to the PolyIran study and TIPS-3^73^ in reducing the risk of MCVE following polypill prescription. Also, polypill reduced ALT level both in patients with fatty liver and those with increased ALT.^72^

 Polypill in a single-tablet form may solve the problem of non-adherence due to multiplicity, unavailability, underprescription, and unaffordability.^74-77^ Aside from clinical efficacy, a primary prevention approach should be cost-effective.^78^ In a systematic review from January 2003 (the onset of polypill concept) to December 2020, the majority of studies emphasized the cost-effectiveness of polypill in CVD prevention.^79^ The components of polypill are widely available in most parts of the world at relatively low costs, and therefore, this combination would be accessible in a cost-effective way for the general population.^80,81^ One of the aims of WHO qualification programs is increasing the availability and affordability of polypill by inclusion of fixed-dose combination therapy in cardiovascular medications.^65^ However, public and private sectors should be encouraged in order to be successful against certain barriers in the way of its implementation such as relative absence of commercial interest.^82^ However, contradictory findings are also reported regarding cost-effectiveness,^83,84^ appropriate indications,^83-85^ and the optimal composition of polypill components.^83,84,86,87^

## Limitations

 In some studies, a considerable amount of data was obtained from death registration systems, and obviously, these types of databases are prone to underreporting and misclassification errors. Also, in some other studies that recruited participants during a definite follow-up period, the small sample size and consequent lack of acceptable statistical power may limit the generalizability of the findings. Also, such studies suffer from certain limitations inherent to prospective investigations.

## Conclusion

 Health planning, policy development, and budget prioritizing need identification of the main causes of PM and its associated risk factors. More than 80% of the global premature deaths happen in LMICs. This is an alarming sign for community health that needs urgent actions. Iran, as a middle-income country, is affected by growing PM and its consequent substantial productivity loss. Health authorities should precisely assess the status of PM in Iran and its underlying causes. The polypill approach helps to prevent thousands of hospitalizations and deaths, and results in decreasing the relevant burden over the next decade.
